# Genus‐Scale Taxonomic Resolution Is Appropriate to Study Coral Demographics: A Case Study With *Pocillopora*

**DOI:** 10.1002/ece3.72180

**Published:** 2025-09-20

**Authors:** Wanchien Victoria Hsiao, Vianney Denis, Stéphane De Palmas, Maria Beger

**Affiliations:** ^1^ School of Biology, Faculty of Biological Sciences University of Leeds Leeds UK; ^2^ Institute of Oceanography National Taiwan University Taipei Taiwan; ^3^ Marine Research Station, Institute of Cellular and Organismic Biology Academia, Sinica I‐Lan County Taiwan; ^4^ Centre for Biodiversity and Conservation Science, School of the Environment University of Queensland Brisbane Queensland Australia

**Keywords:** coral reef, demography, genetic identity, population dynamics, taxonomic sufficiency, vital rates

## Abstract

Coral demography reveals how populations persist, recover and grow, offering key insights into individual responses to environmental variability and their role in shaping reef ecosystems. Taxonomy resolution strongly influences how these dynamics are interpreted, yet fine‐scale distinction among scleractinian species remains unclear. Here, we examine the demographic performance of *Acropora*, *Pocillopora* and *Porites* corals at different taxonomic levels to identify the most appropriate resolution for studying coral population dynamics. In *Pocillopora*, growth did not differ among species or haplotypes, while survivorship varied among species but was also shaped by environmental factors. Comparison between the three genera revealed clear differences in both growth and survival, suggesting that genus‐level and morphological resolution captures sufficient demographic information. Our results highlight the ecological relevance of taxonomic resolution and provide important insights into how population dynamics should be addressed to understand trends and responses to future climate change.

## Graphical abstract

We propose that the appropriate taxonomic resolution should be defined according to the ecological question. Results from our targeted coral population confirm that generalisation at higher taxonomic level provides sufficient demographic information to investigate population and community dynamics and can potentially be used in other comparable systems.

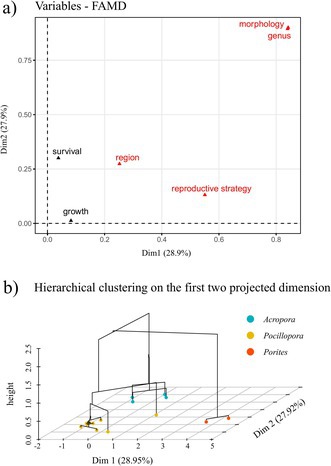

## 1 Introduction

Fine‐scale taxonomic resolution provides insights into the diversity and ecological roles of individuals within ecosystems. It enables us to recognise subtle differences between and within closely related species, improving our understanding of their contributions to community structure and ecosystem functioning (Pacifici et al. 2015). With advanced molecular techniques, fine‐scale genetic resolution at the species, subspecies and haplotype levels can reveal molecular diversity and individual‐specific characteristics (Craft et al. 2010). Such fine‐scale approaches are particularly meaningful for taxa where morphological plasticity or the presence of cryptic lineages complicate species identification (Bickford et al. 2007; Bongaerts et al. 2021). Resolving this hidden diversity allows for the identification of lineage‐specific differences in physiological tolerance, life‐history strategies and ecological interactions, providing insights into how these mechanisms further shape population persistence (Grupstra et al. 2024; Aichelman et al. 2025). On the other hand, climate change and anthropogenic disturbances impose major stressors on ecosystems, increasing the importance of understanding ecological processes from individuals to communities (Bellard et al. 2012; Habibullah et al. 2022). Responses to stressors are not uniform within a community, as variation among different taxonomic levels can lead to different demographic outcomes. For selected species that survived stress events, it remains unclear whether their demographic performance matches that of individuals living in optimal environments.

Given the trade‐off between understanding broad ecological diversity loss and examining responses at the molecular level, there is a strong incentive to investigate the ecological significance of taxonomic resolution. In this aspect, taxonomic sufficiency reflects a compromise between detailed taxonomy and practical ecological assessment, providing enough information to evaluate ecological patterns and processes (Ferraro and Cole 1992; Jones 2008). Consolidating information at higher taxonomic levels generally benefits ecological models with stable data (Arnoldi et al. 2016), whilst modern phylogenetics continues to refine relationships at finer taxonomic levels (Jones 2008; Bennett et al. 2014). However, neglecting ecological differences between closely related species can also lead to a misinterpretation of ecosystem dynamics (Johnston et al. 2021), making taxonomic sufficiency a practical yet controversial approach when assessing community structure and ecological functioning. It is therefore important to evaluate when fine‐scale taxonomic resolution is ecologically meaningful, while recognising that such resolution may be less critical when the primary focus is on ecosystem‐level patterns.

Coral reefs are among the ecosystems most threatened by climate change (Hoegh‐Guldberg and Bruno 2010; Burrows et al. 2011; Eddy et al. 2021). Disturbances such as tropical storms, diseases, and thermal stress‐induced bleaching events are responsible for their biodiversity decline and the loss of structural complexity (Pratchett et al. 2008; Graham and Nash 2013). Climate‐induced thermal stress reveals different susceptibility to bleaching in individuals of different genera, species, haplotypes and even within individual colonies (Burgess et al. 2021; Johnston et al. 2021; Voolstra et al. 2025). Furthermore, lineage or species exhibit high heterogeneity in their biogeographical preferences, including depth, latitude and habitat types (Grupstra et al. 2024). Despite this, lineage or species‐specific demographic performance, such as vital rates of growth, survival and reproduction (Stott et al. 2011; Cant et al. 2020; Capdevila et al. 2020; Mulla et al. 2024), remains overlooked, largely because species distinction is infeasible in the field. Consequently, ecological studies often pool lineages or species, potentially masking differences in vital rates and making it impossible to discern whether observed variation reflects intrinsic biology or environmental influences (Pratchett et al. 2015; Hall et al. 2021; Palacio‐Castro et al. 2023).

In terrestrial ecosystems, population dynamics are mostly studied at the species level, following the traditional concept of a population as groups of individuals belonging to the same species (Berryman 2020). In contrast, coral studies often consider a ‘population’ at a higher taxonomical level, informed by molecular species boundaries and the practicality of taxonomic sufficiency (Bramanti and Edmunds 2016; Kayal et al. 2018; Cant et al. 2020, 2022, 2023). Molecular evidence often confirms species complexes, morphological plasticity and cryptic lineages complicating field identification (Todd 2008; Bongaerts et al. 2021; Oury et al. 2023; Mulla et al. 2024). Pooling coral populations by genus and functional traits can reveal differences in demographic responses following disturbances (Kayal et al. 2018; Cant et al. 2023) and predict changes in population structure and growth variation in future projections (Pisapia et al. 2020). The use of a simplified taxonomy has proven successful in distinguishing corals with different life history strategies and predicting community outcomes under future climate scenarios (Cant et al. 2020). Yet, whether finer taxonomic resolution improves demographic predictions remains unknown, making corals an ideal group in which the ecological relevance of fine‐scaled taxonomy in demography can be studied.

Demographic variation in corals is observed across different levels of taxonomic resolution, from different families, genera, species, to individuals (McWilliam et al. 2022; Lachs et al. 2024). At coarse taxonomic scales, commonly studied families present distinct demographic strategies. Acroporids are often characterised by their rapid growth and high recruitment rates but low survival, and Poritids exhibiting slower growth but higher resistance to stress events (Darling et al. 2012). At finer scales, demographic traits can differ among species within a genus, as individual differences, local environment and symbiont associations further shape performance. Corals within the genus *Pocillopora* are widely distributed throughout the Indo‐Pacific from shallow to mesophotic depths (Pinzón et al. 2013; Dai and Cheng 2020; Johnston et al. 2021; Oury et al. 2023) and are notorious for harbouring cryptic species (Schmidt‐Roach et al. 2013). Although easily identified at the genus level, the presence of morphologically identical but genetically distinct lineages poses a challenge to the study of species‐population and community biology in the field, requiring the use of molecular tools to enable accurate taxonomic classification (De Palmas et al. 2018; Voolstra et al. 2023). The genetic variation within a species complex could lead to morphologically similar cryptic species exhibiting different demographic performances, or contrastingly, morphological similarity may render them demographically similar. The characteristics of the *Pocillopora* complex make it an ideal candidate to uncover the importance of taxonomic resolution and biogeography to demographic processes in corals.

Here, to evaluate the ecological relevance of fine‐scale taxonomy, we aim to determine how the genetic identity of individuals affects their demographic performance by (1) examining how vital rates differ within taxonomic resolution at the genus, species and haplotype levels; (2) distinguishing the relevant importance of biogeography between tropical and subtropical settings by characterising the individual vital rates in contrasting environments; and (3) proposing an appropriate taxonomic resolution for the coral demographics inferred from the case study.

## 2 Methods

### 2.1 Study Sites

**FIGURE 1:**
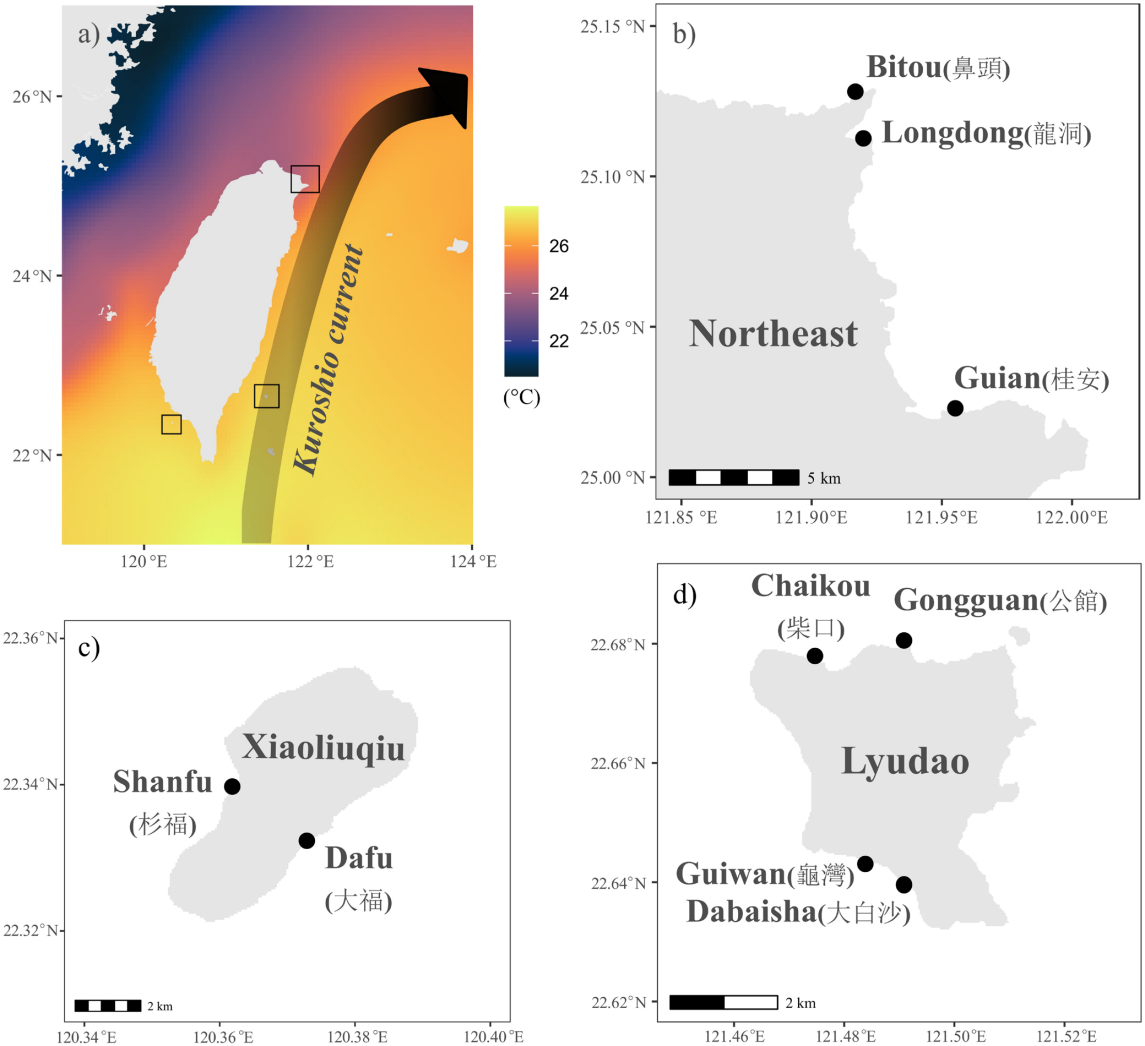
Map of Taiwan and each study region and sites. (a) Location of Taiwan in a transition zone between tropical and subtropical environments, with the selected sites in (b) the subtropical north eastern region; the tropical (c) Xiaoliuqiu island in the southwest and (d) Lyudao in the southeast. Background sea surface temperature in (a) are 30 years' monthly average from 1994 to 2023, derived from NOAA Global Coral Bleaching Monitoring (monthly, 5 km resolution), obtained from NOAA ERDDAP (https://coastwatch.pfeg.noaa.gov).

In Taiwan, *Acropora*, *Pocillopora* and *Porites* live on all coral reefs from south to north (Dai and Cheng 2020) and from shallow to mesophotic depths (Denis et al. 2019). The communities in the south are accreting under tropical conditions, characterised by a relatively high species diversity, with abundant *Pocillopora* that can turn locally dominant (Mulla et al. 2024) and extend to mesophotic depths (De Palmas et al. 2018; Soto et al. 2018). The Kuroshio current passes through the eastern coast, bringing warm and oligotrophic waters northward and towards the Ryukyus archipelago, following the edge of the continental shelf (Dai 2018). The north of Taiwan is located outside the path of the Kuroshio, with corals forming non‐accreting assemblages that are considered marginal compared to the tropical system (Dai and Cheng 2020). The cold China coastal current and northeast monsoon in the winter bring cold waters to the north, resulting in lower SSTs than in other regions at similar latitudes. We selected nine sites in three regions: Guiwan (龜灣), Dabaisha (大白沙), Chaikou (柴口) and Gongguan (公館) in Lyudao (southeast Taiwan); Dafu (大福) and Shanfu (衫福) in Xiaoliuqiu (southwest Taiwan); and Longdong (龍洞), Bitou (鼻頭) and Guian (桂安) in the Northeast (northeast Taiwan) (Figure 1). Monthly mean SSTs ranged between 25.0°C and 30.0°C at Xiaoliuqiu, 25.2°C and 29.6°C around Lyudao, and 18.8°C and 28.1°C on the northern coast (Central Weather Administration of Taiwan, https://www.cwa.gov.tw/). The strong environmental contrasts at a relatively small spatial scale make Taiwan a suitable location to assess the ecological role of fine‐scale taxonomy in the variation of demographic performance (Ribas‐Deulofeu et al. 2016; Denis et al. 2020).

### 2.2 Permanent Plot Monitoring

In 2022, we established ten 1.5 m × 1.5 m permanent plots at 9–12 m depth at each site to record and monitor the diversity and population structure of corals in each region over the long term. For demographic purposes, we specifically targeted areas with a higher abundance of *Pocillopora*, *Acropora*, *Porites* and merulinids. At each site, ten permanent plots were established by attaching numbered cow tags (6 cm × 7.5 cm), either nailed to bare substrate or secured with cable ties within each plot. We plotted out border lines for each plot in the initial survey year to ensure we would be capturing the same area in future surveys. With this method, we managed to find back all permanent plots in subsequent years. We conducted an initial survey of the plots in July 2022 and revisited them in July 2023. For each annual survey, once a plot was relocated, we took a wide‐angle community photo ~3 m above the substrate, aligning it with the laminated reference photo from the previous year to ensure consistent tracking of the same colonies over time. We then zoomed in on each colony within the plots by taking a top‐down close‐up photograph with an Olympus TG‐5 underwater camera and a ruler positioned next to the colony for calibration. For the genetic purpose of this study, we sampled every *Pocillopora* individual larger than 5 cm × 5 cm within our permanent plots (collection permit from the National Science and Technology Council IEC‐R3‐213086). Using a bone cutter, we only collected 3–5 verrucae (2–3 mm^2^), limiting damages to the coral colonies and preserved them in 95% ethanol with their designated numbers for further molecular analysis.

### 2.3 Molecular Taxonomy

We crushed the verrucae in a mortar, then extracted the total genomic DNA using a DNeasy Blood and Tissue Kit (Qiagen) and amplified the mitochondrial Open Reading Frame region using the ‘FATP6.1’ (5′‐TTTGGGSATTCGTTTAGCAG‐3′) and ‘RORF’ (5′‐SCCAATATGTTAAACASCATGTCA‐3′) primers according to Flot et al. (2008). The resulting sequences were edited in BioEdit (Hall 1999) and aligned using the clustalW algorithm implemented in MEGA11 (Tamura et al. 2021) with a final size of 858 bp for each sequence.

We analysed the distribution of haplotypes and the relative dominance of each *Pocillopora* species with a haplotype network using PopART (Leigh and Bryant 2015) and used the Minimal Spanning Network method (Bandelt et al. 1999) to illustrate nucleotide differences between the sequences examined. We obtained references from previously published mtORF haplotype sequences (Pinzón et al. 2013; Hsu et al. 2014; Gélin et al. 2017) to compare and align the results with our sequences. Each *Pocillopora* individual was assigned to a haplotype based on the haplotype network and to a species based on the blast results and morphology.

### 2.4 Demographic Data

For all tracked colonies within the plots, we quantified demographics and genetics of *Pocillopora* and chose *Acropora* and *Porites* for a coarse‐scale demographic comparison due to their common occurrence throughout the study region and their different life history strategies compared to *Pocillopora*. We focused specifically on their vital rates by recording individual changes in size as growth, presence/absence of individual colonies as survival, and new individual corals less than 4 cm^2^ appearing in our permanent monitoring plot as recruits. We determined the colony size by measuring the planar surface area (cm^2^) from photographs using the area calculation in ImageJ and the SizeExtract package in R (Lachs et al. 2022). We then calculated colony growth as the change in planar areas between consecutive years relative to their initial size as an annual percentage growth rate. We also calculated the arithmetic mean radius (AMR) by dividing the planar area by the individual perimeter and calculating the change in the radial extension (Pratchett et al. 2015). Colony survival was determined as the presence/absence of each tracked individual from 2022 to 2023, where we marked corals present in 2022 but not in 2023 as dead. After completing the annotation of the 2022 photographs, we realised some *Pocillopora* colonies within the plots were missed in our 2022 DNA collection, and we resampled them in 2023.

### 2.5 Statistical Analyses

To determine how taxonomic resolution affects the vital rates of individuals, we grouped and compared them between haplotypes, species and genera. To this end, we tested differences in annual percentage growth rates, radial extension and survival rates among and within *Pocillopora* species using analyses of variance (ANOVA). We further conducted Tukey pairwise comparison when ANOVA revealed a significant difference to identify pairs with significantly different vital rates.

Considering *Pocillopora* spp. as a population, we then compared annual growth and survival rates with those of *Acropora* and *Porites* populations and across tropical and subtropical environmental settings. We used Linear Mixed Effects Models (LMMs) to quantify how environmental settings (Xiaoliuqiu and Lyudao being tropical and Northeast being subtropical), study site (nested within environmental settings), species and haplotype affected the annual percent growth and radial extension of *Pocillopora*. We modelled the survival of *Pocillopora* colonies (as a binary response variable) using generalised linear mixed models (GLMMs). The model tested for the effects of initial record size (log transformed) in 2022 and the species identity on *Pocillopora* survival, while we included sites as random factors in the analysis.

Growth and survival rates, along with three additional intrinsic categorical traits related to demographic performance (environmental settings, morphology, reproductive mode), were included in a factor analysis of mixed data (FAMD) and a hierarchical clustering on principal components (HCPC) to measure variation in both continuous and categorical variables. We selected these traits to represent the different environments (tropical vs. subtropical), branching, tabular, sub‐massive morphology and reproductive mode as brooder or broadcast spawners using information from Darling et al. (2012) and/or the coral trait database (Madin et al. 2016). We performed all statistical analyses in R v.4.3.0 (R Core Team 2023) with the packages ‘lme4’ v.1.1.33 (Bates et al. 2015), ‘car’ v.3.12 (Fox and Weisberg 2019), ‘FactoMineR’ v.2.11 (Lê et al. 2008) and ‘factoextra’ v.1.0.7 (Kassambara and Mundt 2020).

## 3 Results

### 3.1 Differences in Vital Rates for *Pocillopora* Haplotypes and Species

**FIGURE 2:**
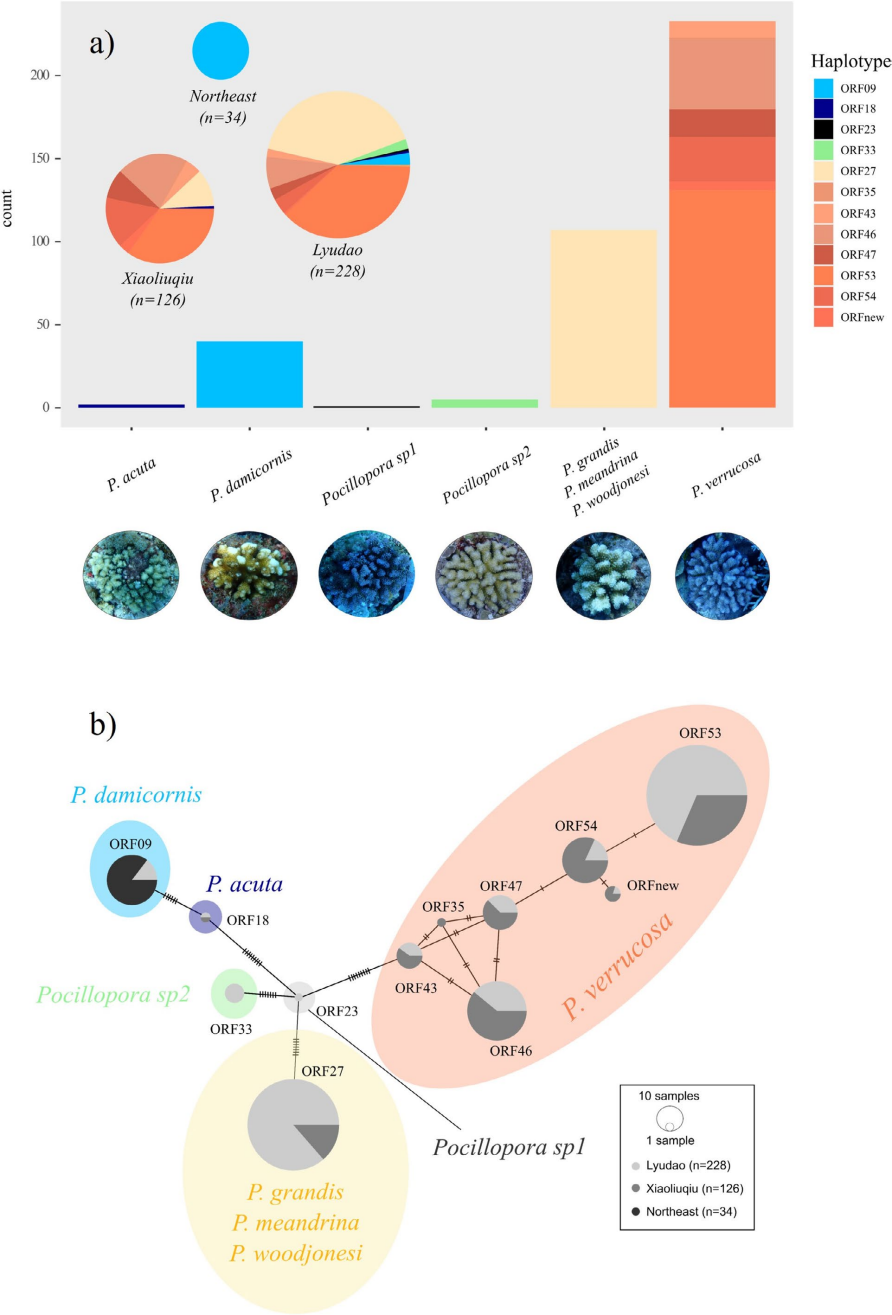
*Pocillopora* haplotypes and species identified in this study. (a) The distribution and typical morphology of each species, and (b) the mtORF haplotype network of *Pocillopora* colonies, visualised by the minimum spanning method; breaks on straight lines connecting haplotypes represent the number of mutations, and species are indicated as coloured fills based on blast results and typical morphologies in a species identification guide (Dai and Cheng 2020). Photos are all taken by Wanchien Victoria Hsiao.

We tracked 388 *Pocillopora* individuals in our permanent monitoring plots between the summers of 2022 and 2023 (Figure 2). We recorded *Pocillopora* species commonly found in Taiwan, including *Pocillopora acuta*, *Pocillopora damicornis* , *Pocillopora grandis*, *Pocillopora meandrina* , *Pocillopora verrucosa* and *Pocillopora woodjonesi* (Dai and Cheng 2020; WoRMS Editorial Board 2025). A total of 315 individuals (81%) survived from 2022 to 2023, with 16 recruits found in 2023. Annual percentage growth rates ranged from −89% to 435% with an average of 15% ± 59% year^−1^ in the north, 35% ± 65% year^−1^ in Xiaoliuqiu and 42% ± 62% year^−1^ in Lyudao (Appendix S1: Table S1). We delineated the tracked *Pocillopora* individuals into eight species and 12 haplotypes. For haplotype ORF27 that harbors more than one species, specimens are further distinguished with the use of morphological characters, such as the overall appearance of the coral morphologies. Thus, *P. meandrina* is distinguished from *P. grandis* and *P. woodjonesi* by its smaller colony size and more uniform and less flattened branches (Dai and Cheng 2020). While we retained the names of the three species to describe this haplotype, most of the analysis later compared demographic traits by haplotype. We observed the highest percentage growth rate in *P. meandrina* (45% ± 65% year^−1^), and the lowest percentage growth rate in *Pocillopora* sp2 (40% ± 50% year^−1^). The radial extension rate had an average of −0.18 ± 0.79 cm year^−1^ in the north, 0.29 ± 0.66 cm year^−1^ in Xiaoliuqiu and 0.06 ± 0.69 cm year^−1^ in Lyudao. It was highest in *P. verrucosa* (0.19 ± 0.70 cm year^−1^) and lowest in *Pocillopora* sp2 (−0.93 ± 0.23 cm year^−1^). Survival rates were lowest in *Pocillopora* sp2 (0.4 ± 0.55) and highest in *P. acuta* and *Pocillopora* sp1, both of which had 100% survival, as two and one individual, respectively, survived the survey period.

**FIGURE 3:**
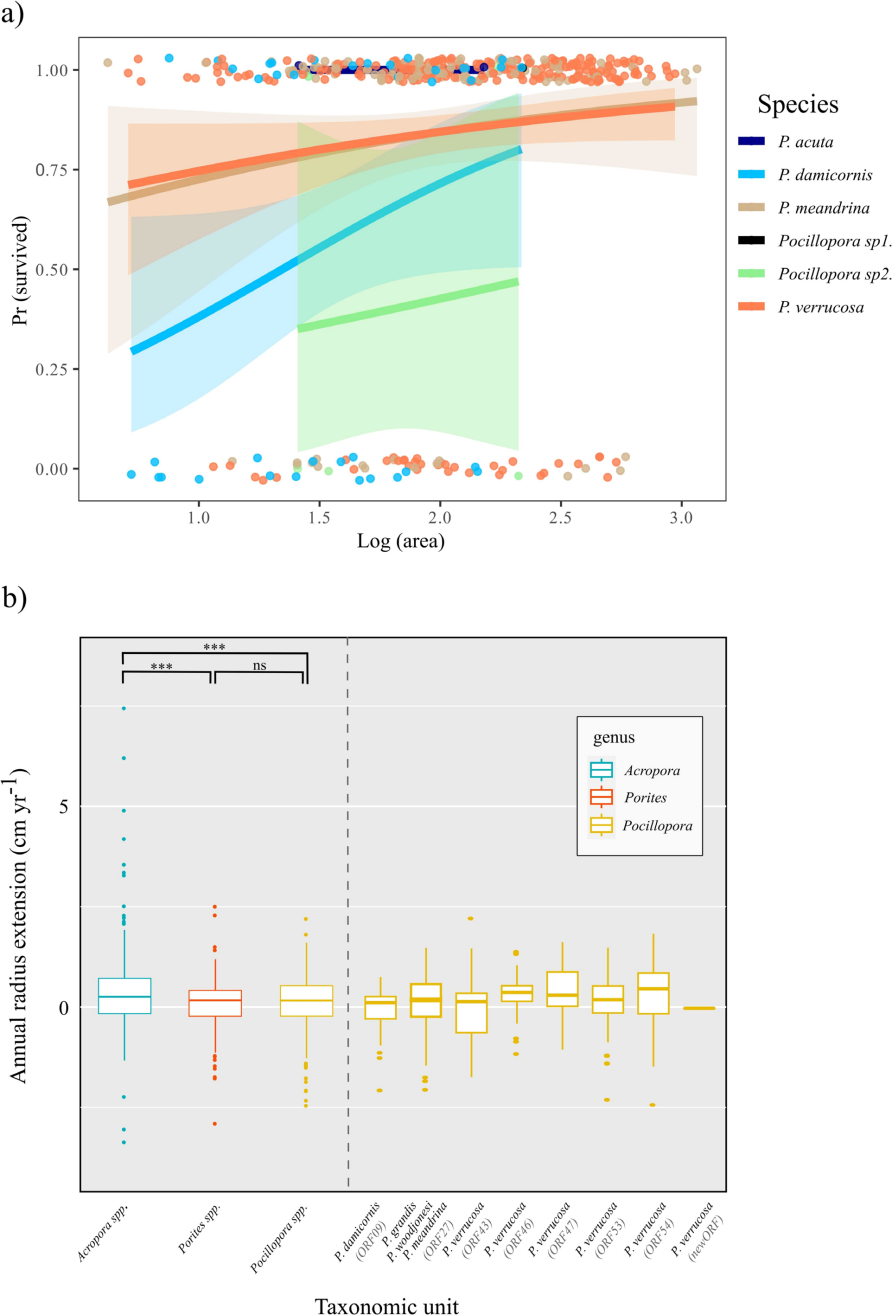
Comparison of growth rates and survival rates among different taxonomic resolution. (a) Annual survival rates across *Pocillopora* species as predicted by log‐transformed colony size and survival classified as 1 = alive and 0 = dead, and (b) annual growth extension of different genera and *Pocillopora* haplotypes, with no significant differences in growth between *Pocillopora* haplotypes.

Excluding species and haplotypes represented by fewer than five individuals, we examined demographic differences in *P. damicornis* , *P. meandrina* and *P. verrucosa* . When comparing between species and haplotypes, annual percentage growth rate (species: ANOVA, *F* = 1.266, *p* = 0.27; haplotype: ANOVA, *F* = 1.397, *p* = 0.17) and extension rates (species: ANOVA, *F* = 1.962, *p* = 0.07; haplotypes: ANOVA, *F* = 1.267, *p* = 0.24) did not show significant differences. In contrast, survival rates differed between species (ANOVA, *F* = 4.286, *p* < 0.001) and haplotypes (ANOVA, *F* = 3.632, *p* < 0.001). Further pairwise comparison suggested differences in *P. damicornis* versus *P. meandrina* and *P. damicornis* versus *P. verrucosa* at the species level; and ORF09‐ORF27 and ORF09‐ORF53 at the haplotype level. The probability of survival in *Pocillopora* spp. increased with colony size (Figure 3a). When comparing the growth and survival rates in different environments, both the percentage growth rate and AMR (ANOVA, *F* = 1.656, *p* = 0.193) showed no significant difference between subtropical and tropical environmental settings, while the survival rate of *Pocillopora* was significantly lower in northeastern Taiwan compared to Lyudao and Xiaoliuqiu (ANOVA, *F* = 13.71, *p* < 0.001). Recruit numbers were low and restricted to only southern regions, so they were not considered in further comparative analysis. Both growth (subtropical: ANOVA, *F* = 3.609, *p* < 0.05; tropical: *F* = 10.41, *p* < 0.001) and survival (subtropical: ANOVA, *F* = 13.44, *p* < 0.001; tropical: *F* = 6.065, *p* < 0.005) showed significant differences among the three genera for populations living in the same environment. Pooling all individuals in both tropical and subtropical environments revealed significant differences in growth (ANOVA, *F* = 10.35, *p* < 0.001, Figure 3b) but not survival (ANOVA, *F* = 2.308, *p* = 0.1) among the three genera. Similar to *Pocillopora* spp., survival was lower for *Acropora* spp. and *Porites* spp. in subtropical northeastern Taiwan than in tropical southern regions.

**FIGURE 4:**
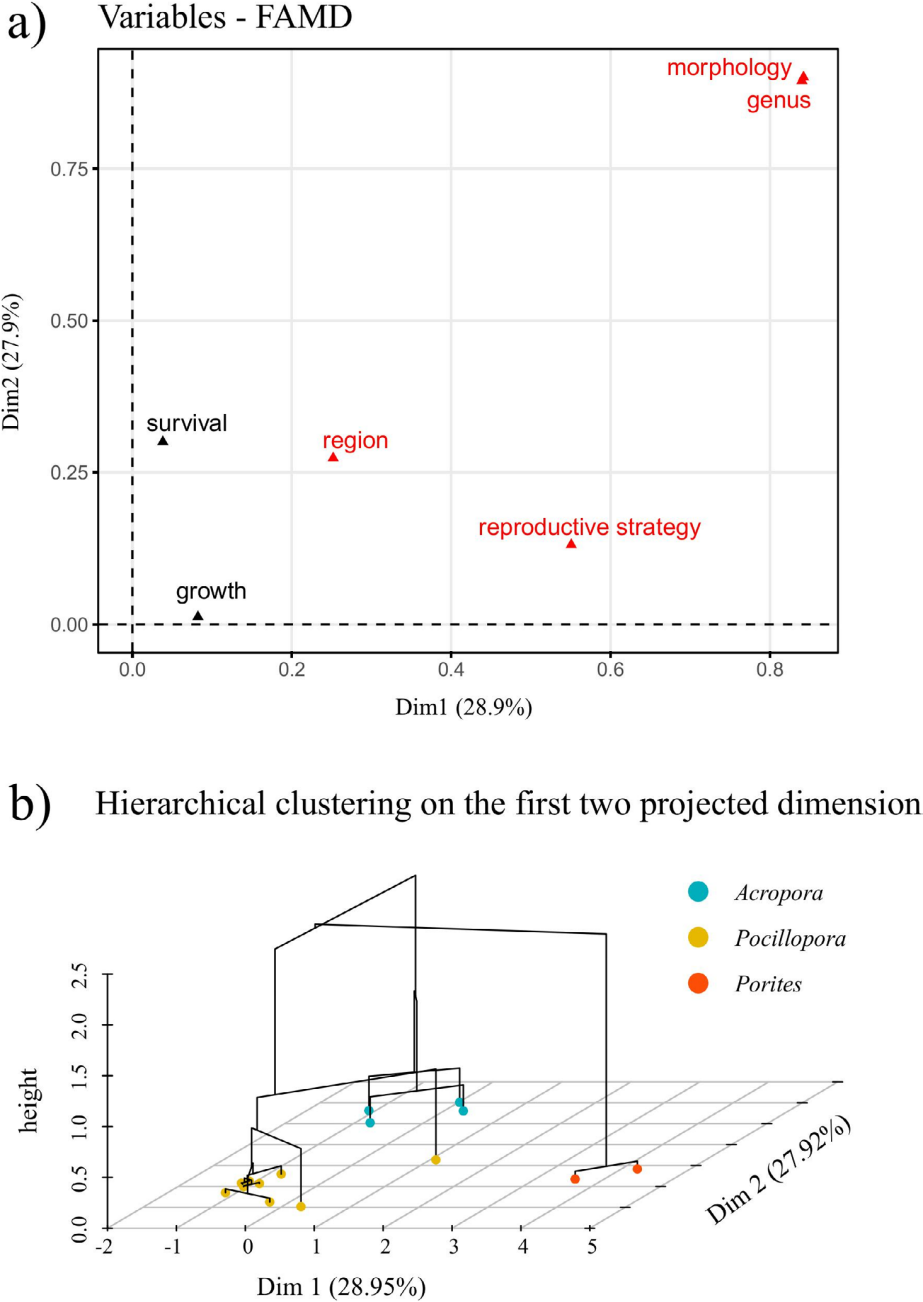
Factor analysis for mixed data (FAMD) projected on a map with the first two components. (a) Contributions of qualitative (red) and quantitative (black) variables on the first and second dimensions. (b) Populations grouped based on their demographic traits. Each point represents one population. Hierarchical clustering on principal component (HCPC) proposed three clusters as the best clustering results, separating the three genera *Acropora*, *Pocillopora* and *Porites*.

More than half of the variance in traits related to demographic performance was explained by the first two axes of the FAMD (PC1: 28.9%, PC2: 27.9%; Figure 4). The different genera are separated along both coordinates. While the different growth forms accounted for most of the variance explained by the first two PCs, the contributions of growth and survival along the first and second PCs were small. The best number of clusters selected by HCPC resulted in three groups separating the three genera *Acropora*, *Pocillopora* and *Porites*.

## 4 Discussion

Understanding the interplay of evolutionary heritage and environmental factors is crucial for assessing and predicting population demographic performance. Here, we find that coral demographic rates vary more among regions and genera than at species and haplotype levels. Growth shows significant differences among different genera, but not within a genus at species and haplotype levels. Yet, the small variation in species survival may indicate a confounding environmental effect, highlighting the need to consider both biotic and abiotic factors when evaluating coral population dynamics. Overall, our results indicate the minor ecological significance of fine‐scale taxonomic resolution in population dynamics compared to large differences among environmental settings and genera. Therefore, we propose working on coral population dynamics at the genus level as a practical and efficient approach to evaluate baseline demographic performance for corals and to study the possible ecological effects of climate change.

Assessing the status of communities with demography is critical, informing the population's boom and bust at different temporal and spatial scales (Edmunds and Riegl 2020). The ecological succession of a *Pocillopora*‐dominated state has been observed in French Polynesia, the Western Pacific and the Great Barrier Reef over the past decades (Adjeroud et al. 2018; Mulla et al. 2024). *Pocillopora* has been observed to form paucispecific communities, where its fast recovery after a disturbance dominates the reef and suppresses the growth and survival of other genera, making it critical for us to understand the dynamics of *Pocillopora* (Dietzel et al. 2020; Mulla et al. 2024). Here, we found no difference in growth among species and haplotypes within *Pocillopora*, but lower growth compared to *Acropora*, suggesting growth is similar within genera but different across genera. The significant difference in survival across regions for *Pocillopora* spp. highlights the influence of the environment, where trade‐offs between demographic traits may result in contrasting demographic performance across tropical reefs and marginal environments (Cant et al. 2023; Nozawa et al. 2021). Higher survival of *Pocillopora*, compared to *Acropora* and *Porites*, partly explains its dominance in tropical environments, leading to *Pocillopora*‐dominated reefs in tropical areas (Forsman et al. 2013; Mulla et al. 2024).

Investigating demographic parameters at species and haplotype levels showed no significant difference at fine‐scale taxonomic resolution, suggesting that variability among species and within species can be comparable. For scleractinian corals, where species boundaries are poorly defined and high intraspecific variation exists in vital rates (Budd et al. 2010), scrutinising individuals to fine‐scale taxonomic resolution may not provide further ecological insights. Our FAMD results showed that including species and haplotype information reduces the variance explained, confirming the redundancy of fine‐taxonomic information for coral demographics. This suggests that coral demographic performance is influenced more by their morphology, physiology and reproductive mode, rather than by fine‐scale genetic identity. In contrast to a study on cryptic lineages of *Acropora hyacinthus* (Gold and Palumbi 2018), we found no differences in growth between *Pocillopora* haplotypes. Unlike *A. hyacinthus* lineages, for which slower growth was associated with robust skeletons, the haplotypes in our study were morphologically indistinguishable. Corals with the same growth form exhibit comparable demographic rates, making morphology a strong predictor of colony growth and mortality (Álvarez‐Noriega et al. 2016; Madin et al. 2016). From our results, we propose that genus and morphology provide sufficient accuracy for demographic studies. Combining these traits gives meaningful and robust information to interpret demographic differences (Darling et al. 2012; Zawada et al. 2019), with morphological features revealing adaptive responses to stress and closely linked to demographic performances (Pratchett et al. 2015; Carlson et al. 2024).

Despite our findings, genetic diversity is crucial for the resilience, adaptation and stability of coral populations (Van Oppen et al. 2015; Drury 2020). Understanding species and haplotype diversity provides insights into genetic variation within a population, which is essential for studying population genetics, evolutionary processes and factors influencing ecosystem resilience (Van Oppen and Gates 2006). Although we found no demographic differences, the distribution of *Pocillopora* species reported here confirms environmental or latitudinal preferences (Figure 2a), with *P. damicornis* dominating subtropical and marginal environments, while *P. verrucosa* , *P. meandrina* and *P. acuta* are more common in tropical regions (Kitano et al. 2015; Poquita‐Du et al. 2017; Fiesinger et al. 2023; Huang et al. 2024). Similarly, *Pocillopora* spp. also show different habitat and depth prevalence and lineage‐specific symbionts among species (Burgess et al. 2021; Johnston et al. 2021, 2022; Voolstra et al. 2023). Physiological traits such as heat tolerance to thermal stress vary among species and lineages (Smith et al. 2017; Burgess et al. 2021; Palacio‐Castro et al. 2023), highlighting the possibility of different bleaching susceptibility, similar to unique responses of individuals, haplotypes and species to pollution (Grupstra et al. 2024). Thus, while broad taxonomic categories can uncover general ecological patterns as we demonstrate here, fine‐scale taxonomic resolution could still be important in many instances to reveal nuances in physiological traits and species distributions.

We confirmed the sufficiency of using higher taxonomic resolution in coral demographics; however, we caution against generalising to coarse taxonomic resolution for all ecological questions. Our results provide observations on short‐term natural dynamics of coral populations that did not experience major disturbances during the experiment, so quantifying the effect of environmental variation in stress events could still provide further information on how populations respond to changes. Collecting both genetic and demographic data is labour and cost‐intensive, especially for marine studies, such that our results represent dynamics on a short time scale and a relatively limited spatial scale. Given the above challenges, a comprehensive individual trait data set associated with both environmental and genetic effects on the demographic performance of corals is much needed. Incorporating genetic and demographic information with existing open‐access trait databases will have the advantage of including more individuals and traits, improving our understanding of trade‐offs between different life‐history traits, which could be relevant to refining demographic projection models and to better understanding the effect of environmental filtering (Sommer et al. 2014).

Reconciling fine‐scale taxonomic resolution with its ecological importance involves understanding the level of taxonomic detail necessary to answer specific ecological questions. While fine‐scale taxonomic resolution can provide individual/species‐specific information for accurately assessing their physiological responses to disturbances, here we show that coarse taxonomic groups at the genus and morphological level provide a general understanding of community dynamics and ecosystem functioning that can be sufficient for ecological questions. Our study provides a ‘baseline’ demographic performance of coral populations during a regular year without stress events. We consider documenting demographic performance during a regular year equally important, as it allows for assessment of how genotypes selected under stress events perform outside of stress periods. With the improvement of genetic tools, phylogenetics is constantly refining species boundaries, and the past definition of coral species may now be incorrect (Budd et al. 2010; Quattrini et al. 2019; Oury et al. 2025). To obtain relevant data gathered from the field, it is important to find the level at which genetic differentiation becomes important in ecology. The decision of how much taxonomic detail to use depends on the ecological context, the research question, and the available resources for species and lineage identification. For coral population dynamics, our results demonstrated that in the absence of stress, generalising individuals at a coarse taxonomic level yields sufficient information to investigate the demographic performance of different populations. This finding endorses other studies that have either considered ‘populations’ as species within the same genus, or species categorised as the same morpho‐functional group (Kayal et al. 2018; Cant et al. 2020; Logan et al. 2021). Thus, understanding coral reef ecosystem dynamics from a coarse taxonomic level can offer significant ecological insights to provide a practical and impactful way to evaluate and manage coral reef ecosystems under the pressures of climate change.

## Author Contributions

**Wanchien Victoria Hsiao:** conceptualization (equal), data curation (lead), formal analysis (lead), methodology (equal), writing – original draft (lead), writing – review and editing (equal). **Vianney Denis:** conceptualization (equal), formal analysis (supporting), funding acquisition (equal), methodology (equal), resources (equal), writing – original draft (supporting), writing – review and editing (equal). **Stéphane De Palmas:** conceptualization (equal), methodology (equal), writing – review and editing (equal). **Maria Beger:** conceptualization (equal), funding acquisition (equal), resources (equal), supervision (lead), writing – original draft (supporting), writing – review and editing (equal).

## Conflicts of Interest

The authors declare no conflicts of interest.

## Supporting information


**Data S1:** ece372180‐sup‐0001‐supinfo.docx.

## Data Availability

The data and code used in this manuscript can be found at the following link: https://github.com/wcvhsiao/Genus‐Scale‐Taxonomic‐Resolution‐Is‐Appropriate‐to‐Study‐Coral‐Demographics‐ECOL‐EVOL.
