# Supplementary material for: Genus‐Scale Taxonomic Resolution Is Appropriate to Study Coral Demographics: A Case Study With Pocillopora
Source: Ecol Evol. 2025 Sep 20;15(9):e72180. doi: 10.1002/ece3.72180 (PMC12449659; doi:10.1002/ece3.72180)
Supplement: Supplementary file 1 — Data S1: ece372180‐sup‐0001‐supinfo.docx. [file ECE3-15-e72180-s001.docx]

**Table S1. Summary of Haplotype demographics found in current study corresponding to literature references.**

| Species | Haplotype names  from previous studies | Recorded location from previous studies | Percentage  Growth rate  (yr^-1^) | Arithmetic Mean Radius  (cm) | Survival rate | Region | | |
| --- | --- | --- | --- | --- | --- | --- | --- | --- |
|  |  |  |  |  |  | Northeast | Xiaoliuqiu | Lyudao |
| *Pocillopora damicornis* | ORF09 (Gélin et al. 2017)  Type 4a (Pinzón et al. 2013) | Taiwan, Australia, Hawaii, Japan, Moorea, New Caledonia | 0.25±0.63 | -0.09±0.70 | 0.58±0.5 | 34 | 0 | 6 |
| *Pocillopora acuta* | ORF18 (Gélin et al. 2017)  Type 5a (Pinzón et al. 2013) | Taiwan, Andaman, Australia, Hawaii, Kenya, Madagascar, Mauritius, Mayotte, Moorea, New Caledonia, Palau, Reunion Island, | -0.28±0.53 | -0.36±0.24 | 1.00 | 0 | 1 | 1 |
| *Pocillopora sp1.* | ORF23 (Gélin et al. 2017)  Type 8a (Pinzón et al. 2013) | Taiwan, Moorea, New Caledonia | 0.19 | 0.04 | 1.00 | 0 | 0 | 1 |
| *Pocillopora sp2.* | ORF33(Gélin et al. 2017) | Taiwan | -0.39±0.50 | -0.93±0.23 | 0.40±0.54 | 0 | 0 | 5 |
| *Pocillopora meandrina*  *Pocillopora grandis*  *Pocillopora woodjonesi* | ORF27 (Gélin et al. 2017)  Type 1a (Pinzón et al. 2013) | Taiwan, Andaman, Australia, Galapagos, Hawaii, Moorea, New Caledonia, Palau, Reunion Island | 0.46±0.64 | 0.07±0.70 | 0.85±0.36 | 0 | 14 | 92 |
| *Pocillopora verrucosa* | ORF43 (Gélin et al. 2017)  Type 3g (Pinzón et al. 2013) | Taiwan, Arabian Gulf, New Caledonia, Red sea, Reunion island | 0.35±0.56 | 0.08±1.15 | 1.00 | 0 | 6 | 4 |
| *Pocillopora verrucosa* | ORF35 (Gélin et al. 2017)  Type 3h (Pinzón et al. 2013) | Taiwan, New Caledonia, Red sea | NA | NA | 0 | 0 | 1 | 0 |
| *Pocillopora verrucosa* | ORF46 (Gélin et al. 2017)  Type 3a (Pinzón et al. 2013) | Taiwan, Australia, Galapagos, Hawaii, Madagascar, New Caledonia, Palau, Reunion Island | 0.51±0.67 | 0.29±0.54 | 0.81±0.40 | 0 | 26 | 16 |
| *Pocillopora verrucosa* | ORF47 (Gélin et al. 2017)  Type 3b (Pinzón et al. 2013) | Taiwan, Australia, Galagapos, New Caledonia | 0.21±0.43 | 0.39±0.76 | 0.82±0.39 | 0 | 11 | 6 |
| *Pocillopora verrucosa* | ORF54 (Gélin et al. 2017)  Type 3d (Pinzón et al. 2013) | Taiwan, Andaman, Eastern Australia, New Caledonia, Palau | 0.56±0.98 | 0.23±1.00 | 0.85±0.36 | 0 | 19 | 8 |
| *Pocillopora verrucosa* | New from this study | Taiwan | -0.06±0.62 | -0.03±0.01 | 0.40±0.55 | 0 | 4 | 1 |
| *Pocillopora verrucosa* | ORF53 (Gélin et al. 2017)  Type 3f (Pinzón et al. 2013) | Taiwan, Eastern Australia, New Caledonia | 0.34±0.53 | 0.15±0.62 | 0.88±0.33 | 0 | 44 | 88 |

Gélin, Pauline, Bautisse Postaire, Cécile Fauvelot, and Hélène Magalon. 2017. "Reevaluating species number, distribution and endemism of the coral genus Pocillopora Lamarck, 1816 using species delimitation methods and microsatellites." *Molecular Phylogenetics and Evolution* 109: 430-446.

Pinzón, Jorge H, Eugenia Sampayo, Evelyn Cox, Leonard J Chauka, Chaolun Allen Chen, Christian R Voolstra, and Todd C LaJeunesse. 2013. "Blind to morphology: Genetics identifies several widespread ecologically common species and few endemics among Indo‐Pacific cauliflower corals (Pocillopora, Scleractinia)." *Journal of Biogeography* 40 (8): 1595-1608.
